# Ligation-free ribosome profiling of cell type-specific translation in the brain

**DOI:** 10.1186/s13059-016-1005-1

**Published:** 2016-07-05

**Authors:** Nicholas Hornstein, Daniela Torres, Sohani Das Sharma, Guomei Tang, Peter Canoll, Peter A. Sims

**Affiliations:** Department of Systems Biology, Columbia University Medical Center, New York, NY 10032 USA; Columbia University M.D./Ph.D. Program, Columbia University Medical Center, New York, NY 10032 USA; Graduate Ph.D. Program in Pharmacology and Molecular Signaling, Columbia University Medical Center, New York, NY 10032 USA; Department of Pathology and Cell Biology, Columbia University Medical Center, New York, NY 10032 USA; Department of Neurology, Columbia University Medical Center, New York, NY 10032 USA; Department of Biochemistry and Molecular Biophysics, Columbia University Medical Center, New York, NY 10032 USA; Columbia Sulzberger Genome Center, Columbia University Medical Center, New York, NY 10032 USA

**Keywords:** Ribosome profiling, Brain, Translation

## Abstract

Ribosome profiling has emerged as a powerful tool for genome-wide measurements of translation, but library construction requires multiple ligation steps and remains cumbersome relative to more conventional deep-sequencing experiments. We report a new, ligation-free approach to ribosome profiling that does not require ligation. Library construction for ligation-free ribosome profiling can be completed in one day with as little as 1 ng of purified RNA footprints. We apply ligation-free ribosome profiling to mouse brain tissue to identify new patterns of cell type-specific translation and test its ability to identify translational targets of mTOR signaling in the brain.

**Electronic supplementary material:**

The online version of this article (doi:10.1186/s13059-016-1005-1) contains supplementary material, which is available to authorized users.

## Background

Ribosome profiling allows genome-wide measurements of ribosomal occupancy with single-nucleotide resolution [1]. Using deep sequencing as a readout for protein synthesis, the technique has enabled the discovery of previously unannotated open reading frames (ORFs) [1–4] and provided new insights into the mechanisms of translation initiation and elongation[5], localized translation [6], and the signaling pathways underlying translational control [7, 8]. In addition, ribosome profiling has been applied in many cellular contexts, including yeast [1], bacteria [9], primary mammalian cells [2], and complex tissues [10], to assess the role of translational control in basic physiological processes and its dysregulation in diseases like cancer.

**Fig. 1:**
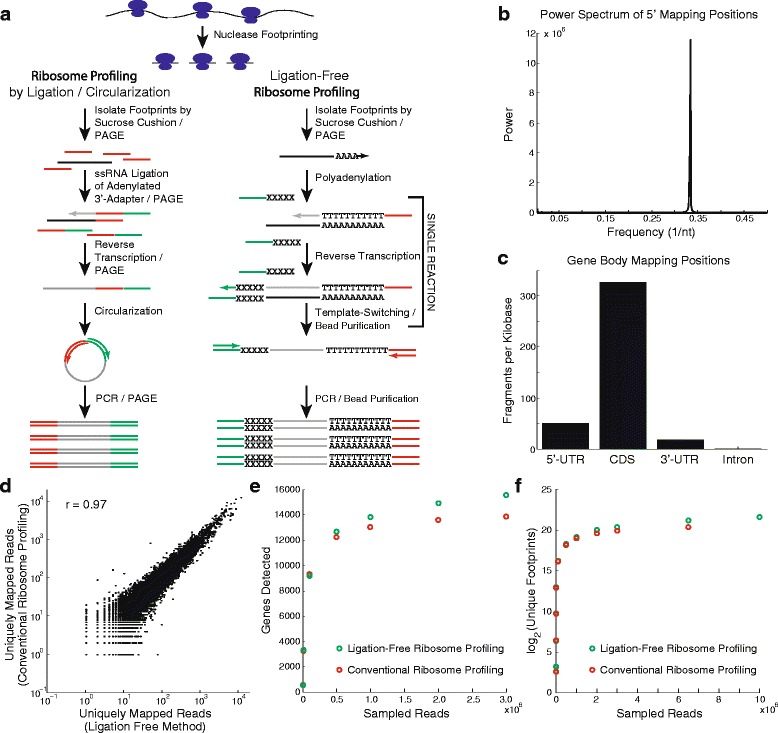
Comparison of ligation-free ribosome profiling with conventional methods. **a** The steps involved in conventional ribosome profiling and ligation-free ribosome profiling. **b** The power spectrum of 5′ mapping positions from coding sequence (*CDS*) reads resulting from the ligation-free ribosome profiling method shows clear three-base periodicity that is characteristic of ribosome profiling libraries and reflects the single-codon translocation of the ribosome. **c** Gene body distribution of mapped reads from ligation-free ribosome profiling show strong preference for CDS, an additional property inherent to ribosome profiling libraries. **d** Comparison of the number of uniquely mapped reads per gene in libraries generated with footprints from mouse forebrains prepared with a conventional ribosome profiling strategy and the ligation-free method; the Pearson correlation r = 0.97 indicates a concordance between the two methods. **e** Saturation analysis showing the number of unique genes detected following downsampling of ligation-free ribosome profiling and conventional ribosome profiling. **f** Saturation analysis showing the number of unique footprints detected following downsampling of ligation-free ribosome profiling and conventional ribosome profiling. *PAGE* polyacrylamide gel electrophoresis, *PCR* polymerase chain reaction, *ssRNA* single-stranded RNA, *UTR* untranslated region

While ribosome profiling is widely used, the library preparation procedure is relatively complex [11]. Most protocols involve nuclease footprinting of polysomal RNA followed by purification of ribosome-bound mRNA footprints using a sucrose gradient, sucrose cushion, or gel filtration column. After isolation of mRNA footprints by gel electrophoresis, one of multiple library preparation schemes is used to attach universal sequence adapters to the mRNA or cDNA footprints using either single-stranded intermolecular ligation [12, 13] and/or intramolecular circularization [1, 11] (Fig. 1a). Because these protocols often involve multiple ligation, gel purification, and nucleic acid precipitation steps, library preparation alone typically takes several days [11]. Here, we report a new approach to library construction for ribosome profiling that eliminates ligation and requires only one initial gel purification step to isolate RNA footprints (Fig. 1a). The procedure, which is based on template switching [14, 15], is highly sensitive and requires only ~1 ng of gel-purified RNA footprints. Following footprint isolation, library construction for ligation-free ribosome profiling can be completed in one day.

In addition to characterizing the performance of ligation-free ribosome profiling, we applied our technique to assess cell type-specific translational regulation in the murine brain. The brain harbors a broad diversity of cell types, including astrocytes, oligodendrocytes, microglia, glial progenitors, endothelial cells, and many different types of neurons that likely control translation through different signaling pathways. In addition, many neuron-specific transcripts are translated locally in dendrites and translational control has been shown to play a key role in memory [16–19]. We took advantage of a recently reported database of neural cell-specific gene expression [20] to identify patterns that indicate cell type-specific regulation of translation. As an orthogonal validation for neuron-specific genes, we used the RiboTag system [21] to purify and identify actively translated transcripts from excitatory neurons in the cortex of Camk2a-Cre/RiboTag mice. Finally, we used our technique to identify the genes controlled by mammalian target of rapamycin (mTOR) signaling in the brain by conducting ribosome profiling on the brains of mice treated with AZD-8055, an ATP-competitive inhibitor of mTOR that crosses the blood–brain barrier [22, 23].

## Results

### A ligation-free protocol for ribosome profiling

Ribosome profiling is more complicated than conventional RNA-Seq because the ribosome-protected mRNA footprints are short (~30 nucleotides) and lack poly(A) tails, which are often used as handles for either isolation or reverse transcription of eukaryotic mRNA. Previously established protocols for ribosome profiling address this problem by single-stranded ligation of a universal adapter to the 3′ end of mRNA footprints to facilitate reverse transcription, which incorporates a longer adapter into the 5′ end of the resulting cDNA [11]. Intramolecular ligation (circularization) of the cDNA effectively attaches a universal adapter to the 3′ end of the cDNA to enable PCR enrichment of the library [11]. Alternatively, a second ligation reaction can be used to attach an adapter to the 3′ end of the cDNA. These ligation reactions are notoriously inefficient and require excess adapter, which is typically removed by gel purification and subsequent overnight precipitation of the product [11]. These multi-step procedures and intermediate purification steps require multiple work days, are intrinsically lossy, and, therefore, require relatively high input [11].

To address these issues, we have applied the template-switching approach to library construction that has been successfully implemented in other low-input RNA sequencing protocols such as single-cell RNA-Seq [24–26]. Specifically, we have adapted a newly developed version of the SMARTer library construction technology (Clontech) for ribosome profiling (Fig. 1a). We first polyadenylate dephosphorylated RNA footprints using RNA poly(A) polymerase, similar to the earliest reported protocol for ribosome profiling [1]. We then reverse transcribe the polyadenylated footprints using an enzyme with template-switching activity. In a template-switching reaction, the reverse transcriptase (RT) first extends a primer (in this case oligo(dT) linked to a universal sequence on its 5′ end) to produce cDNA. Once the RT reaches the end of the RNA template, the terminal transferase activity intrinsic to the RT adds a low complexity sequence to the 3′ end of the cDNA in a non-template-directed fashion. The reaction is carried out in the presence of a second universal sequence adapter that is 3′ terminated with a low-complexity sequence, which hybridizes to the tail added to the cDNA by the RT. Upon hybridization of this second sequence adapter, the RT switches templates and copies the second adapter onto the 3′ end of the cDNA. As a result, both 5′ and 3′ universal adapters are simultaneously added to the cDNA in a single reaction without single-stranded ligation or intermediate purification steps. We then deplete the resulting product of rRNA using complementary oligonucleotides [11] and enrich the deep sequencing library by PCR.

### Comparison of ligation-free ribosome profiling with conventional ribosome profiling

We used ligation-free ribosome profiling to measure genome-wide translation in the forebrains of adult mice. Unlike fragments generated in RNA-Seq, ribosome footprints map to the transcriptome with a three-nucleotide periodicity due to the characteristic translocation interval of the ribosome as it translates codons [1]. To verify that the RNA libraries generated using our technique originate from ribosome footprints, we computed the power spectrum of the 5′ mapping positions of RNA fragments (Fig. 1b). As expected, the data are highly periodic with a characteristic frequency of ~0.33 nucleotides^−1^, similar to what has been observed for conventional ribosome profiling [1]. In addition to three-nucleotide periodicity, ribosome profiling also exhibits a characteristic gene body distribution. The majority of reads are expected to map to the coding sequences (CDSs) of transcripts, whereas relatively few should map to the untranslated regions (UTRs) [1]. Many genes have been shown to contain unannotated upstream ORFs (uORFs) and so we also expect that more reads will map to the 5′ UTRs than the 3′ UTRs, which are largely depleted of ribosomes. As shown in Fig. 1c, ligation-free ribosome profiling reads map to the transcriptome with the expected gene body distribution.

To further validate the technique, we compared these results with our previously reported mouse forebrain data that we generated using conventional ribosome profiling [10]. Figure 1d shows that the ribosome footprint counts for each gene across the two data sets are highly correlated. We also compared the gene detection efficiency, saturation properties, and library complexities of the two data sets. We note that in our previously reported experiment with conventional ribosome profiling, we used more input monosomal RNA for library construction than in the current experiment with ligation-free ribosome profiling. In Fig. 1e, f, we use downsampling analysis to show that the two data sets are quite similar in terms of both the number of genes detected and number of unique ribosome footprints detected, respectively, at a given sequencing depth. These results imply that the library complexities produced by the two protocols are highly comparable.

In order to determine the sensitivity of both conventional and ligation-free ribosome profiling, we generated libraries from a defined 34-base RNA oligonucleotide at five input levels ranging from 0.01 to 100 ng. We constructed Illumina libraries from each dilution using the convention ribosome profiling protocol described by Ingolia et al. [11] and the ligation-free protocol described here. We then assessed our yield for each dilution using an Agilent Bioanalyzer (Additional file 1: Figure S1). We found that the ligation-free method is more sensitive and able to generate detectable libraries from less than 1 ng of input. For both methods we were able to generate quantifiable libraries; however, we were only able to generate libraries at 10 and 100 ng of input when using the conventional protocol with nine PCR cycles. In contrast, we were able to generate detectable libraries at all concentrations tested when using the ligation-free protocol with nine PCR cycles. We note that the 10 and 100 ng input libraries made with the ligation-free protocol exhibit over-amplification as evidenced by a broader product length distribution at higher-than-expected molecular weights. To directly compare all of the samples, we kept the number of PCR cycles constant and note that lower cycle numbers could be used to avoid over-amplification of higher input libraries with the ligation-free protocol. In addition, we note higher cycle numbers may result in sufficient library yields for the conventional protocol at lower concentrations, although this could result in increased amplification bias.

### Cell type-specific translation in the brain

One of the key metrics obtainable from ribosome profiling experiments is the translation efficiency (TE), which can be computed for each gene as the ratio of its ribosome footprint density to its expression level measured by RNA-Seq [1]. TE is proportional to the number of ribosomes per transcript averaged over all copies of a given gene.

**Fig. 2:**
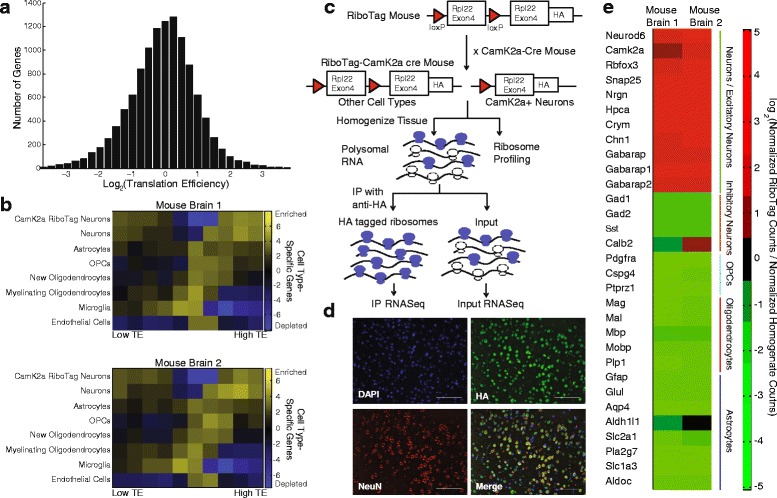
Unique patterns in the translation efficiency of cell type-specific genes in the brain. **a** The broad range of translation efficiencies (TEs) across genes expressed in the mouse brain based on ligation-free ribosome profiling. **b** TEs measured in two different mouse brains with ligation-free ribosome profiling were combined with cell type-specific RNA-Seq data to systematically associate cell type-specific gene expression and TE. We used gene set enrichment analysis (GSEA) to associate gene sets assembled from genes with similar TEs with a ranked list of all genes ordered by cell type-specificity for each cell type in the brain. The resulting heatmaps show the enrichment of genes with different TEs in cell type-specific genes for each cell type. Cell type-specific genes were identified using either RNA-Seq data from sorted populations or RiboTag RNA-Seq data (for Camk2a-expressing neurons). *OPC* oligodendrocyte precursor cell. **c** The RiboTag mouse model shows how the Camk2a-RiboTag mouse was generated. This provides an orthogonal means of identifying neuron-specific genes that are actively translated. *HA* hemagglutinin, *IP* immunoprecipitation. **d** Fluorescence imaging shows that Rpl22-HA (from the RiboTag allele) expression is specific to Rbfox3+ (NeuN+) cells (a pan-neuronal marker). **e** Heatmap of the RiboTag enrichment scores following immunoprecipitation of polysomes from Camk2a-RiboTag mouse brains demonstrates strong enrichment of genes specific to excitatory neurons and depletion of genes specific to other cell types in the brain in two different mouse brains

We used ligation-free ribosome profiling and RNA-Seq to measure TE in the brain of an adult mouse, a complex tissue comprised of many different cell types. Both ribosome footprint densities and expression levels are complicated by cellular composition. This is also true to a large extent for TE; however, because TE is a ratio, the TE measured in homogenized tissue for a cell type-specific gene is accurate for both the tissue and the specific cell type that expresses the gene. Figure 2a shows the broad distribution of TEs for genes expressed in the brain of an adult mouse. While this result implies that there is a great deal of translational regulation in the brain, it tells us nothing about the contributions of different cell types.

We validated our TE measurements by performing quantitative PCR (qPCR) on a set of highly translated (Syt1, Snap25) and lowly translated (Trpv6, Tgfb1, Pkd1) genes based on our ribosome profiling data. We first used sucrose gradient fractionation to separate mRNAs based on the number of bound ribosomes and collected fractions. We then used qPCR to assess the relative abundance of each gene in each fraction (Additional file 2: Figure S2). Several complications are associated with directly comparing qPCR data obtained from polysome profiles and ribosome profiling data. While the majority of transcripts for a highly translated gene may appear in polysomes with more than five ribosomes per transcript, resolution constraints make it difficult to accurately measure the number of bound ribosomes for each fraction, particularly for heavier polysomes. Furthermore, calculating TE based on log ratios without correcting for cytosolic mRNA levels has been previously shown to produce an inaccurate estimation of TE [27]. While it is difficult to quantitatively compare TE calculated from next-generation sequencing with that obtained from qPCR, we found that the highly translated genes probed are clearly shifted to heavier polysomes compared with the lowly translated genes probed. For example, we found that the maximum abundance of the highly translated genes Syt1 and Snap25 were in the seventh and ninth polysome fractions (greater than five ribosomes per transcript), respectively (Additional file 2: Figure S2). However, the maximum abundances of Trpv6, Tgfb1, and Pkd1, all of which are lowly translated, were in the fourth and fifth fractions (two or three ribosomes per transcript).

We also compared our ligation-free ribosome profiling and RNA-Seq data with a previously published whole-brain mass-spectrometry data set obtained from a mouse of similar genetic background and age [28]. We found that our ribosome profiling data were better correlated with protein abundance in the brain than our corresponding RNA-Seq measurements (Additional file 3: Figure S3). Hence, some of the difference in the explained variance may be attributable to the contribution of translation regulation on protein expression. This result is consistent with previously published observations in yeast in which mass spectrometry, RNA-Seq, and ribosome profiling were compared [1].

A recent study by Zhang and colleagues [20] produced RNA-Seq expression profiles from seven different cell types in the brain by sorting or immune-panning, including astrocytes, neurons, oligodendrocyte progenitor cells (OPCs), newly formed oligodendrocytes, myelinating oligodendrocytes, microglia, and endothelial cells. We used this data set to compute cell-type enrichment scores proportional to the specificity with which each gene is expressed in each cell type (see “Methods”). We then divided the transcriptome into ten gene sets evenly binned by TE and conducted gene set enrichment analysis (GSEA) against rank-ordered lists of cell-type enrichment scores for each cell type [29]. This analysis allowed us to systematically associate genes with varying degrees of cell type specificity and TE. The normalized enrichment score (NES) for each GSEA is shown in the heatmap in Fig. 2b (with bin-by-bin and cell type-by-cell type statistical analysis in Additional file 4: Figure S4), which reveals several interesting patterns. First, we found that microglial genes generally exhibit low TEs. Because we are studying the brains of healthy mice, these microglia are presumably not in an activated state. Previous studies have shown that protein synthesis-associated pathways are upregulated in microglia in certain disease contexts [30] and so these results could be dependent on genotype or other activating conditions such as injury or an inflammatory stimulus. Conversely, neurons, when considered as a broad group, exhibit the highest degree of variation in TE among their cell type-specific genes. As shown in Fig. 2b, most neuronal genes are either very highly or very lowly translated, suggesting that neuronal genes are under a relatively high degree of translational regulation in comparison with other cell types in the brain.

Translational control is well-known to play an important role in neuronal function and memory formation. Structurally, neurons are highly complex cells that make extensive use of local translation to efficiently modulate protein expression far from the soma [31]. To validate our observation that neuronal genes are highly translationally regulated, we used the RiboTag system to isolate polysomal mRNAs from a specific neuronal subtype, namely excitatory neurons that express Camk2a. As shown in Fig. 2c, the RiboTag mouse harbors a modified ribosomal protein L22 (Rpl22) gene with a floxed terminal exon followed by a second copy of the terminal exon with a triple hemagluttinin tag (HA-tag) [21]. We crossed the RiboTag mouse with a mouse that expresses Cre recombinase under the control of the Camk2a promoter to produce mice which express HA-tagged ribosomes in Camk2a-expressing cells. Figure 2d shows that, as expected, the HA-tag is expressed exclusively in neurons, marked here by the pan-neuronal marker NeuN (Rbfox3). Hence, we can isolate polysomes from homogenized brain tissue of Camk2a-RiboTag mice and purify mRNA–ribosome complexes that originate from Camk2a-expressing neurons by immunoprecipitation (IP) of the HA-tag (Fig. 2c). We obtained RNA-Seq expression profiles from both homogenized brain tissue and immunoprecipitated polysomes of two Camk2a-RiboTag mice. We compared the expression levels of each gene in the immunoprecipitated and homogenate profiles and observed that canonical markers of excitatory neurons were enriched by IP, whereas markers of other cell types in the brain, including inhibitory neurons, were depleted by IP (Fig. 2e). We then repeated the GSEA described above with TE gene sets and genes rank-ordered based on their enrichment by RiboTag IP. This analysis recapitulated the results found for neuronal genes derived from purified neurons in that genes specific to Camk2a-expressing neurons, and not just neurons in general, appear highly translationally regulated (Fig. 2b). A subset of genes expressed in these neurons exhibit relatively high TE, while the remaining exhibit relatively low TE. Not only do these results provide an orthogonal validation of our GSEA based on pan-neuronal gene expression, they also show that the pattern holds for a specific subtype of excitatory neurons in the cerebral cortex.

Finally, these data reveal a simple developmental trend in the oligodendrocyte lineage. Oligodendrocytes, which are primarily responsible for enwrapping neuronal axons with myelin sheaths, are a unique cell type in that their progenitor cells (OPCs) are widely distributed in the adult brain, where they actively proliferate and differentiate to generate new myelinating oligodendrocytes. Hence, we can detect gene expression and translation from different stages of oligodendrocyte development within homogenized brain tissue. Based on our analysis, OPC-specific genes are translated more efficiently than those of either newly formed or mature, myelinating oligodendrocytes, which exhibit the lowest TE of the three. As shown in our statistical analysis in Additional file 4: Figure S4, the comparison between OPCs and myelinating oligodendrocytes is very significant for highly translated genes, as is the comparison between newly formed oligodendrocytes and myelinating oligodendrocytes. While one might expect myelinating oligodendrocytes to be less translationally active in comparison with OPCs because they are post-mitotic, their primary role in the brain is to produce large amounts of myelin, which is comprised mainly of proteins and lipids. Nonetheless, we found that most myelin genes have low TE compared with the overall median in the brain (log_2_(TE) = −0.02), including Mog (−0.15), Mbp (−0.51), Mobp (−1.42), and Mag (−0.28), with the exception of the transmembrane protein Plp1, which has a TE of 1.02. Hence, despite the importance of protein synthesis to the function of myelinating oligodendrocytes, translation of oligodendrocyte-specific genes is relatively inefficient.

**Fig. 3:**
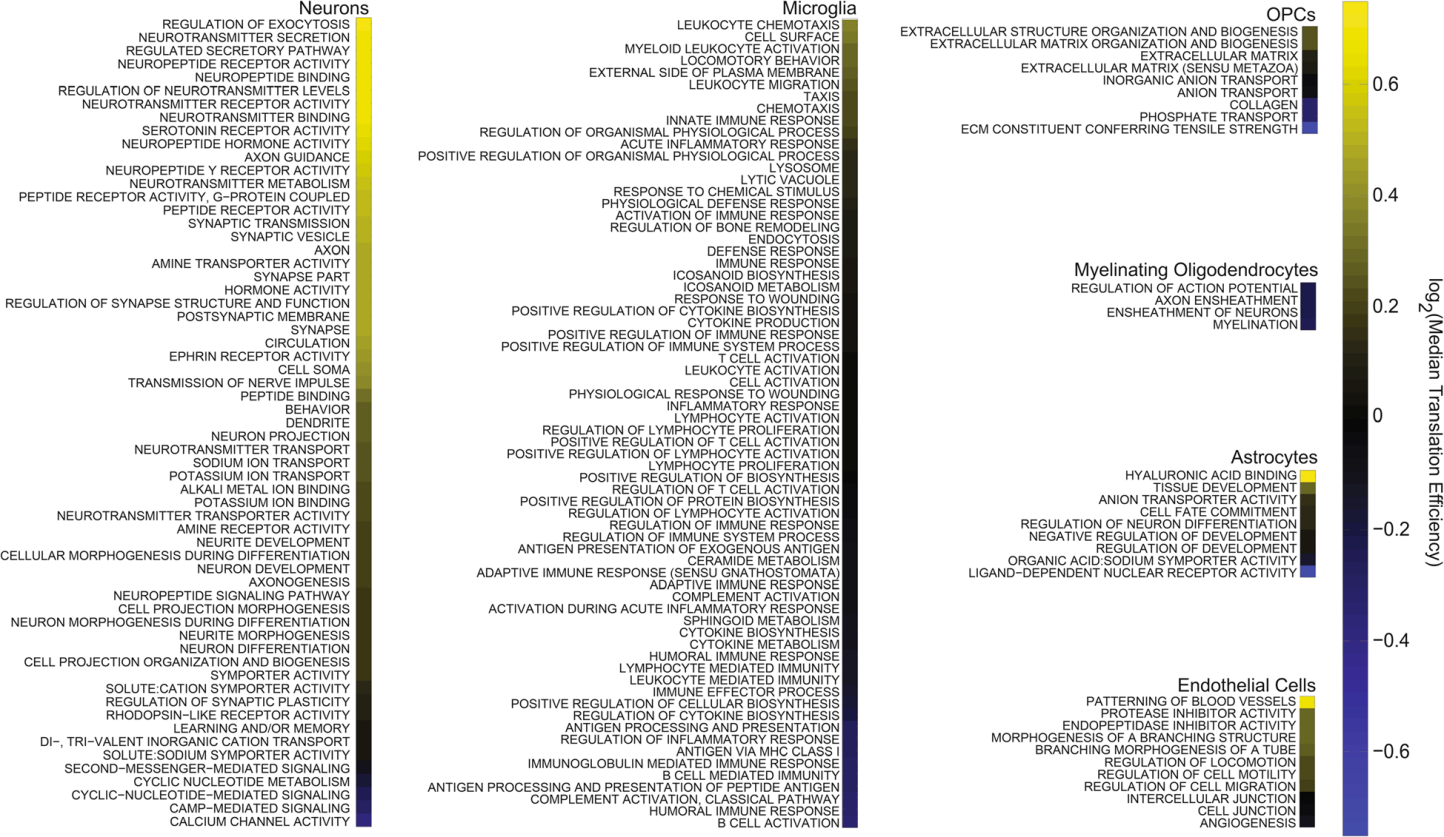
Cell type-specific gene ontologies recapitulate global translation efficiency trends. We used GSEA to identify gene ontologies enriched in cell type-specific genes. An enrichment score was calculated for all genes in each cell type based on RNA-Seq data from sorted neural cell types. This information was placed into six different rank lists, one for each cell type. A gene ontology was defined as being cell type-specific if it had a NES score for a cell type that was at least three units greater than the next highest NES score. Ligation-free ribosome profiling datasets from two mouse brains were averaged and used to calculate the median translation efficiency for each ontology. Highly enriched ontologies and their median translation efficiencies in descending order are displayed in the heatmaps

We used gene ontologies (GOs) to further refine these insights into cell type-specific translation. In Fig. 3, we used GSEA to identify GOs that were strongly associated with cell type-specific genes from each of six cell types in the brain (Additional file 5: Table S1). We then produced heatmaps indicating the median TE of each GO. Figure 3 contains many of the qualitative patterns found in Fig. 2b, with neuronal GOs exhibiting a broad range of TEs and microglial and oligodendrocyte GOs exhibiting relatively low TEs. In addition, this analysis reveals some of the gene functions associated with the highly translated and lowly translated neuronal genes. For example, genes associated with synaptic function, particularly those that are released by neurons in a synapse, are generally highly translated. Conversely, sodium, potassium, and, most particularly, calcium channels exhibit much lower TEs.

### uORFs and 5′ UTRs in the brain

**Fig. 4:**
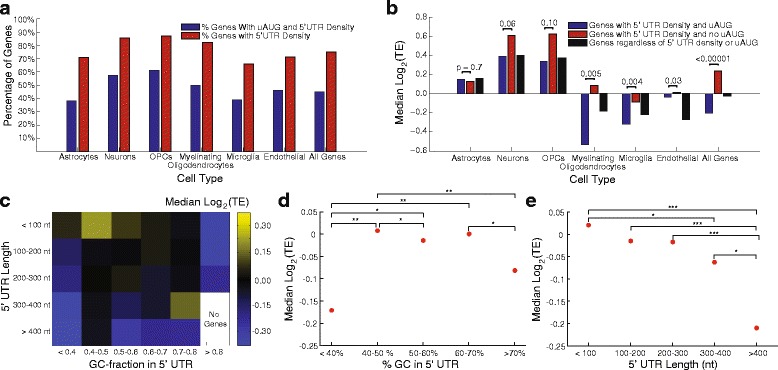
Features of 5′ UTRs are associated with CDS translation. **a** The percentage of cell type-specific genes with at least one ribosome footprint mapping to their 5′ UTR is plotted together with the percentage of cell type-specific genes with 5′ UTR ribosomal density and also containing a uAUG sequence. These values are highly consistent across cell types. **b** Genes containing a uAUG and 5′ UTR ribosomal density had lower CDS TE compared with genes without a uAUG. This effect was consistent across multiple cell types and was significant for myelinating, microglial, and endothelial cells. Furthermore, this effect was seen regardless of cell-type specificity. **c** Heatmap showing the relationship between 5′ UTR GC content, 5′ UTR length and CDS TE. Very high and very low GC content are associated with lower median TE. As the length of the 5′ UTR increases, the median TE of the CDS decreases. **d**, **e** The relationships between GC content (**d**) and 5′ UTR length (**e**) are independently plotted against median TE for each length or GC-content bin; *p ≤ 0.05, **p ≤ 0.01, ***p ≤ 0.001

One of the most intriguing findings of ribosome profiling studies in eukaryotes is the prevalence of unannotated uORFs which manifest as ribosomal density in the 5′ UTRs of mRNAs [1–4]. Recent studies have further refined these observations using computational methods to infer which instances of 5′ UTR density actually represent active uORF translation and correlate with direct observations of specific peptides in mass spectrometry [4]. Using our mouse brain dataset produced with ligation-free ribosome profiling, we have investigated the 5′ UTR ribosomal density among cell type-specific genes. Figure 4a shows that we detect 5′ UTR ribosomal density in a consistent fraction of genes across all cell type-specific gene sets. Previous studies using conventional ribosome profiling have shown that 5′ UTR ribosomal density is associated with different levels of CDS translation depending on sequence context [3, 10, 32]. Specifically, 5′ UTRs that harbor ribosome density but do not contain AUG sequences are associated with genes with higher TE in the annotated CDS, suggesting a potential regulatory role for upstream ribosomal density. Figure 4b shows that this general trend is borne out across all of our cell type-specific gene sets.

We also sought to determine how more general features of the 5′ UTR affect translation efficiency of the corresponding CDS in the brain. Figure 4c is a heatmap that simultaneously displays the relationships between CDS TE and both the length and GC content of the 5′ UTR across the transcriptome. Figure 4d, e display these relationships independently. In general, longer 5′ UTRs are associated with low TE and both high and low GC content are associated with low TE. Previous studies have shown that genes with highly structured 5′ UTRs are less abundant at the protein level in yeast [33], which is consistent with the reduced TE associated with long, GC-rich 5′ UTRs observed here.

### Translational targets of mTOR in the brain

A common application of ribosome profiling is the identification of translational alterations in response to perturbations such as drug treatment or stress. Cells have evolved elegant mechanisms for regulating the translation of specific genes, often through the interaction of signaling molecules with translation factors that control TE through specific *cis*-regulatory elements in mRNA. We sought to further test the efficacy of our ligation-free ribosome profiling method in the context of this important application by identifying the translational targets of mTOR signaling in the brain.

mTOR plays a crucial role in the translational control of ribosomal proteins and protein factors involved in translation initiation and elongation [34]. Many of these genes contain a terminal oligopyrimidine (TOP) motif in their 5′ UTRs through which translational control is thought to be mediated [34]. Multiple studies have used ribosome profiling to show that mTOR inhibition causes a coherent decrease in the TEs of the TOP motif-containing genes in cell culture [7, 8]. mTOR is an important drug target in multiple neurological disorders [35]. For example, rapalog inhibitors of mTOR have been shown to mitigate seizures in certain contexts [36]. We sought to determine whether mTOR controls the same set of target genes in brain.

**Fig. 5:**
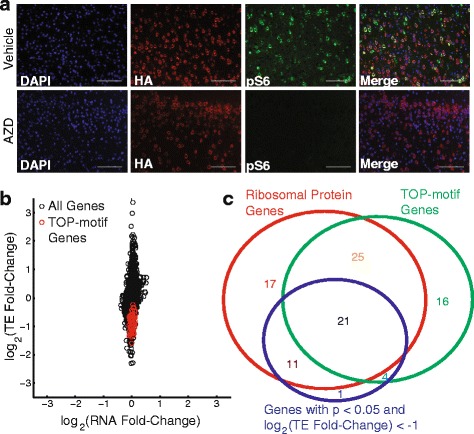
mTOR controls TOP motif-containing genes in the brain. Camk2a-RiboTag mice were treated for 1 h with the ATP-competitive mTOR inhibitor AZD-8055 and were used to generate ligation-free ribosome profiling libraries from brain tissue and fluorescence imaging data. **a** Treatment for 1 h with AZD-8055 was sufficient to drastically decrease levels of phosphorylated Rps6 in mouse brains. HA-staining indicates the presence of HA-tagged Rpl22 (RiboTag) in cells expressing Camk2a. **b** Comparison of RNA and TE fold changes between AZD-8055-treated and untreated mice. TE exhibits larger amplitude changes than RNA levels in response to mTOR inhibition in the brain. The TE of TOP motif-containing genes are greatly reduced. **c** We used RiboDiff to identify genes with significant differential translation efficiency and DESeq2 to identify genes with significant differential RNA expression in treated versus untreated mice. The Venn diagram shows the overlap between genes with significant translational reduction after AZD-8055 treatment, ribosomal proteins, and TOP motif-containing genes

We treated mice for 1 h with AZD-8055, an ATP-competitive inhibitor of mTOR that has been shown to cross the blood–brain barrier [22, 23]. We used a competitive inhibitor because previous work has shown that allosteric mTOR inhibitors like rapamycin do not induce the same level of translational alterations as competitive inhibitors [8]. This is, in part, because allosteric compounds do not fully inhibit 4E-BP phosphorylation, which is thought to be the primary mediator of translational control through which mTOR acts [7]. Figure 5a shows the effects of AZD-8055 on the phosphorylation of Rps6, which is phosphorylated by the protein kinase Rps6kb1 (i.e., p70S6K), which is activated by mTOR. As expected, Rps6 phosphorylation is clearly detectable in the brain, particularly in neurons, in an untreated mouse but becomes undetectable in a mouse treated with AZD-8055 based on both immunofluorescence (Fig. 5a) and western blot analysis (Additional file 6: Figure S5).

We used ligation-free ribosome profiling to compare genome-wide TEs in mice treated with AZD-8055 and vehicle-treated mice. We then conducted a differential TE analysis comparing the treated and untreated conditions to identify genes with significant translational alterations (see “Methods”). Figure 5b shows that, overall, the amplitude of the observed alterations in TE are much larger than those found at the level of transcription alone. In addition, Fig. 5b shows that all of the canonical TOP motif-containing genes exhibit reduced TE in the brains of mice treated with the mTOR inhibitor AZD-8055. Furthermore, most of these TE changes are highly significant based on our differential translation analysis (Fig. 5c). Overall, we found 37 genes with significant TE reduction after treatment and fold change amplitudes greater than 2. Of these 37 genes, 25 were in the list of canonical TOP motif-containing genes [7]. Of the remaining 12 genes, all but one are ribosomal proteins and all 12 genes clearly contain TOP motifs (Additional file 7: Table S2). Not only do these results further validate our ligation-free ribosome profiling technique, they also demonstrate rapid and widespread translational control of the TOP motif-containing genes by mTOR in the brain only 1 h following administration of an inhibitor.

## Discussion

We have demonstrated a new approach to library construction for ribosome profiling and used it to show new cell type-specific patterns of protein synthesis in the brain. Through the use of template switching, we bypassed several inefficient and time-consuming steps associated with conventional ribosome profiling, such as ligation, and eliminated almost all gel purification steps. Using ligation-free ribosome profiling, we can construct libraries from as little as 1 ng of purified RNA footprints and the resulting library complexity and gene detection efficiency are comparable to those of conventional ribosome profiling. Furthermore, due to the elimination of several enzymatic and precipitation steps, the amount of time required to perform library construction with ligation-free ribosome profiling is as little as one day following isolation of RNA footprints.

Although ligation-free ribosome profiling offers the advantages described above, conventional ribosome profiling has some advantages in terms of resolving ribosome footprints. Both the 3′ and 5′ ends of ligation-free ribosome profiling reads are associated with low complexity sequences. Specifically, the 3′ end is poly(dA) and the 5′ end is another low complexity sequence. This complicates precise determination of the ribosome footprint insert sequence, a problem that is resolved by ligation of specific sequence adapters in the conventional library construction protocol. Nonetheless, for the purposes of measuring translation efficiency and other metrics presented here, this shortcoming does not pose a major issue.

Using ligation-free ribosome profiling, we have shown that genes expressed in specific cell types exhibit distinct distributions of translation efficiency in the brain. Interestingly, most neuron-specific genes have either relatively high or low translation, implying that they are under a high level of translational regulation. We validated these findings in Camk2a-expressing neurons using the RiboTag system, which allows isolation of polysomal mRNA from specific cell types. At the level of GOs, neuron-specific genes involved in synaptic function are efficiently translated as a group compared with, for example, neuron-specific ion channels. We also found that genes associated with three stages of oligodendrocyte differentiation exhibited different translation efficiencies. OPC-specific genes were translated more efficiently than genes specific to newly formed oligodendrocytes, while fully differentiated, myelinating oligodendrocyte-specific genes had the lowest translation efficiency of the three stages. We have also determined the relationship between CDS translation efficiency and the GC content and length of 5′ UTR sequences in the brain. In general, long, GC-rich 5′ UTRs are associated with low translation efficiency, consistent with the notion that genes containing highly structured 5′ UTRs are lowly translated. Finally, we observed widespread translational repression of genes containing the TOP motif in response to mTOR inhibition. Our treatment window was just 1 h, suggesting that these alterations comprise the earliest effects of competitive mTOR inhibition in the brain.

## Conclusions

Taken together, the above results provide convincing evidence that ligation-free ribosome profiling allows rapid and quantitative translational profiling, even in complex tissues like the mammalian brain. We anticipate that the simplified procedure described here will expand the use of ribosome profiling and may enable new, low-input or larger-scale applications.

## Methods

### Camk2a-RiboTag mouse model

Camk2a-cre mice (JAX ID 005359) have the mouse calcium/calmodulin-dependent protein kinase II alpha (*Camk2a*) promoter driving Cre recombinase expression in the forebrain, specifically in principal excitatory neurons. Camk2a-cre mice were crossed to RiboTag mice (JAX ID 011029) which contain a conditional knock-in allele where exon 4 of the ribosomal protein L22 (Rpl22) is flanked by loxP sites, followed by an identical exon tagged with three repeated hemagglutinin epitope coding sequences (HA-tag). The resulting Camk2a-cre-RiboTag cross expresses the HA-tagged Rpl22 protein in principal excitatory neurons. Camk2a-cre heterozygotes were crossed to homozygous RiboTag mice and genotyped with primers for Cre (GCG GTC TGG CAG TAA AAA CTA TC (transgene), GTG AAA CAG CAT TGC TGT CAC TT (transgene), CTA GGC CAC AGA ATT GAA AGA TCT (internal positive control forward), GTA GGT GGA AAT TCT AGC ATC ATC C (internal positive control reverse)) and for RiboTag (GGG AGG CTT GCT GGA TAT G (forward), TTT CCA GAC ACA GGC TAA GTA CAC (reverse)).

Previous reports have shown that recombination with the Camk2a promoter-driven cre begins during the third postnatal week and is completed by the fourth postnatal week; therefore, we chose to use mice that were 3 months old for all experiments [37].

### Drug delivery and tissue collection

AZD-8055 (Selleckchem) was dissolved in Captisol and diluted to a final Captisol concentration of 30 % (w/v). A single dose of AZD-8055 was administered by oral gavage (100 mg/kg). Vehicle consisted of 30 % captisol and was also delivered by oral gavage. Camk2a-cre-RiboTag mice were sacrificed 1 h after AZD-8055 or vehicle administration; two mice were used per condition. Cervical dislocation was performed and the right frontal lobe of the brain was collected and snap-frozen in liquid nitrogen prior to polysome extraction. The remaining brain lobes were fixed in 4 % paraformaldehyde for 48 h and embedded in paraffin for histological analysis.

### Immunofluorescence

Fixed brains were embedded in paraffin and tissue sections (5 μm) were used for staining. To remove excess paraffin, slides were immersed in xylene then rehydrated by incubation in 100, 95, and 75 % ethanol. Slides were washed in phosphate-buffered saline (PBS) then water. For antigen retrieval 10 mM citrate buffer (pH 6.0) was heated and slides were immersed for 20 minutes, followed by PBS washes. Sections were then permeabilized with 0.5 % Triton-X100 in PBS for 15 minutes, blocked in 5 % goat serum for 1 h, and incubated with primary antibodies overnight at 4 °C. Sections were washed three times in PBS and incubated with AlexaFluor-conjugated secondary antibodies (1:1000, Invitrogen) for 1 h at room temperature and counterstained with DAPI. Stained tissue sections were imaged using a Nikon TE2000 epifluorescence microscope.

### Antibodies

The following primary antibodies were used for immunofluorescence and western blotting: mouse monoclonal anti-HA.11 ascites (1:500, Biolegend #901515), rabbit anti-pS6 S240/244 (1:500, Cell Signaling #2215), rabbit anti-NeuN (1:500, Cell Signaling #12943), rabbit anti-pS6 S235/236 (1:1000, Cell Signaling #2211), rabbit anti-S6 (1:1000, Cell Signaling #2217), rabbit anti-β-actin (1:1000, Cell Signaling #4970S). The following secondary antibodies were used for immunofluorescence and western blotting: goat anti-rabbit Alexa 488 (1:1000, Invitrogen #A11008) and goat anti-mouse Alexa 568 (1:1000, Invitrogen #A11031).

### Western blot analysis

Tissue was collected 1 h after vehicle or AZD-8055 administration (20 mg/kg or 100 mg/kg AZD-8055). The right frontal brain lobe was lysed from male mice that were 12 weeks old. Tissue was lysed in 1 mL cell extraction buffer (Invitrogen #FNN10011) supplemented with protease (Sigma #P7626) and phosphatase inhibitors (Sigma#P5726, #P0044) with a Dounce homogenizer. Lysate was centrifuged and the supernatant was collected for total protein quantification. Total protein (30 μg) was loaded to a NuPAGE 4-12 % Bis-Tris gel and subject to gel electrophoresis according to the manufacturer’s instructions (Invitrogen #NP0321BOX). Bands were detected by fluorescent imaging using the Typhoon imaging system.

### Tissue processing for RNA

Snap frozen tissue samples (5 mg) were homogenized at 4 °C with a Dounce homogenizer in 1 mL of polysome lysis buffer (20 mM Tris-HCl pH 7.5, 250 mM NaCl, 15 mM MgCl_2_,1 mM DTT, 0.5 % Triton X-100, 0.024 U/ml TurboDNase, 0.48 U/mL RNasin, and 0.1 mg/ml cycloheximide). Homogenates were centrifuged for 10 minutes at 4 °C, 14,000 × g. The supernatant was removed and used for the isolation of ribosome footprints, total RNA, and polysome immunoprecipitation (IP). SUPERase-In (0.24U/mL) was added to the lysate used for polysome IP to prevent RNA degradation.

### Polysome IP

Lysate (100 μL) was used as the input, from which RNA was extracted using the RNeasy Mini Kit (Qiagen). The remaining lysate was used for indirect IP of polysomes. We coupled 15 μL of mouse monoclonal anti-HA.11 (ascites, Biolegend) to lysate with rotation at 4 °C for 4 h. We used 150 μL of protein G-coated Dynabeads (30 mg/mL, Life Technologies) and washed them with 600 μL polysome lysis buffer three times. The conjugated lysate was then added to protein G-coated Dynabeads and incubated with rotation at 4 °C overnight. Beads were then washed three times with 500 μL of polysome lysis buffer. RNA was extracted from magnetic beads with polysome release buffer (20 mM Tris-HCl pH 7.3, 250 mM NaCl, 0.5 % Triton X-100, 50 mM EDTA) four times for 5 minutes each (140 μL × 4). RNA from the pooled supernatants (560 μL) was then extracted with the RNeasy Mini Kit (Qiagen) and RNA integrity was assessed using a Bioanalyzer (Agilent).

### RNA sequencing libraries

RNA samples were provided to the Columbia Sulzberger Genome Center for poly(A)-selection and RNA-Seq using the Illumina TruSeq kit. A total of four RNASeq libraries were generated for AZD-treated and vehicle control mice. RNASeq libraries were generated from matched samples used in ligation-free ribosome profiling experiments. Four additional libraries were sequenced from non-ribosome profiling matched samples; two total input samples and two matched HA-IP samples.

### Polysome profiling and qPCR validation

The left frontal lobe, contralateral to the portion used to generate a ligation-free ribosome profiling library, was conserved and used to generate qPCR data from polysome profiles. The tissue sample was lysed with a Dounce homogenizer, as previously described, and fractionated with a 15–50 % sucrose gradient at 37,000 RPM for 3.5 h. Polysome profiles were obtained and RNA was extracted from fractions using an RNA Clean and Concentrator column (Zymo). cDNA was generated with a high-capacity RNA to cDNA kit (Life Technologies). qPCR was performed on each fraction with five probes representing genes with either high or low TE as found by ribosome profiling: SYT1 (Mm00436858_m1), SNAP25 (Mm01276449_m1), TGFB1 (Mm01178820_m1), PKD1 (Mm00465434_m1), and TRPV6 (Mm00499069_m1) (ThermoFisher). TaqMan Universal Master Mix (Life Technologies) was used to setup qPCR reactions and a Bio-Rad CFX-96 was used to amplify and read plates. All experiments were performed in triplicate. CQ was determined for each sample and an average CQ number was calculated for each set of triplicates. CQ numbers were converted using abundance = 2^1−CQ^ and the highest value for each gene normalized to 1.

These values were then plotted according to the polysome peak from which they were obtained.

### Ribosome Profiling Sensitivity Measurement

A 34-base RNA oligo, ‘AUGUACACGGAGUCGAGCUCAACCCGCAACGCGA[Phos]’, was purchased from Sigma and used to generate conventional and ligation-free ribosome profiling libraries. Conventional libraries were generated using the protocol described in Ingolia et al. [11] using the primers described in Gonzalez et al. [10]. The template oligo was serially diluted to the following concentrations; 100 ng, 10 ng, 1 ng, 0.1 ng and 0.01 ng. Following dephosphorylation, both conventional and ligation-free construction schemes were used to attempt to generate libraries at each concentration. For the final PCR step for all libraries in both protocols, PCR was restricted to 9 cycles with 90 % of the remaining material. Samples were diluted as necessary and assessed with a High-Sensitivity DNA Bioanalyzer Chip (Agilent).

### Poly(A) tailing of size selected fragments

Ribosomal footprints were isolated with a sucrose cushion, size-selected, and dephosphorylated as previously described [2, 11]. Following dephosphorylation of size-selected footprints, we determined the concentration of input material using a Bioanalyzer (RNA 6000 Pico Chip, Agilent Technologies). We found that quantification with a Bioanalyzer was more accurate than with a RNA Qubit or Nanodrop due to the presence of Glycoblue (Ambion) as a precipitant. We used a newly developed kit for small RNA library construction (SMARTer® smRNA-Seq Kit for Illumina®, Clontech catalog number 635030) to generate ligation-free ribosome profiling libraries. Between 1 and 5 ng of size-selected material was used as input and diluted with water to a total volume of 7 μL. Ensuring that reagents remained on ice, polyadenylation mix was prepared by combining 7 μL of RNA input with 2.5 μL of mix 1, which includes poly(A) polymerase. After adding the polyadenylation mix, samples were incubated for 5 minutes at 16 °C. Following incubation, samples were immediately placed on ice to ensure the poly(A) tailing reaction did not continue.

### Reverse transcription and template switching

Proceeding from the previous step within 5 minutes, samples were allowed to cool for 1 minute on ice. A 3′ smRNA dT primer (1 μL) was added to each tube and mixed by pipetting. Samples were incubated for 3 minutes at 72 °C and then transferred to ice for 2 minutes. During this incubation step, RT master mix was prepared. The RT master mix consisted of 6.5 μL smRNA mix 2, 0.5 μL RNase inhibitor, and 2 μL PrimeScript RT and 9 μL was added to each sample and mixed by pipetting. Samples were placed in a thermocycler pre-heated to 42 °C and incubated at 42 °C for 1 h followed by a 10-minute incubation at 70 °C to heat-inactivate the enzyme.

### Ribosomal RNA depletion

Ribosomal RNA (rRNA) was depleted from samples with a subtraction oligo pool as described previously [11]. Briefly, the subtraction oligo pool consists of several dozen short biotinylated oligos complementary to rRNA fragments that commonly contaminate mammalian ribosome profiling libraries. Following hybridization, the oligos are removed with magnetic streptavidin beads. We combined 10 μL of the previous RT reaction with 2 μL of the subtraction oligo pool and mixed. The mixture was heated to 100 °C for 90 s in a thermocycler. Following heating, the mixture was placed into a 100 °C heatblock and allowed to cool to 37 °C. Upon reaching 37 °C, the mixture was removed from the heatblock and incubated for 15 minutes at 37 °C in a thermocycler. While the depletion mixture incubated, 37.5 μL myOne Strepavidin C1 DynaBeads (Invitrogen) were prepared for each sample. Streptavidin beads were washed three times with an equal volume of 1× polysome buffer. Following the final wash, beads were split into 25 μL and 12.5 μL aliquots. After removing the polysome buffer from the 25 μL aliquot of beads, the depletion mixture was added to the beads and the resulting mixture was incubated for 15 minutes at 37 °C. The depletion mixture was then recovered from the beads using a magnet and added to the second, 12.5 μL aliquot of beads. The resulting mixture was incubated for 15 minutes at 37 °C. Ensuring no beads were carried over, the depleted RT reaction was then recovered using a magnet.

### PCR library amplification

The SeqAmp DNA polymerase included in the SMARTer® smRNA-Seq Kit (Clontech) was used to amplify cDNA from the depleted RT reactions. For the experiments reported, we used the low-throughput primer set from Clontech (catalog number 634844) but have also had success using Clontech’s high-throughput primers (included in the SMARTer® smRNA-Seq Kit). PCR reactions were incubated for 1 minute at 98 °C followed by 12 cycles of a two-step protocol of 98 °C for 10 s and 68 °C for 10 s.

### Purification of libraries

Purification is necessary due to the presence of primers and other contaminants from upstream reactions. Furthermore, it is critical to ensure reduction of a non-product secondary peak ~25 nucleotides smaller than the product peak. The secondary peak increases linearly with PCR cycle number and is inversely related to total input used. Because the secondary peak is similar to the expected peak size from ribosome profiling and can interfere with sequencing, it is essential to ensure that it is at least less than half the size of the product-peak. We performed two rounds of purification with AMPure XP beads (Beckman Coulter) at a 1.8× and 1.2× ratio (due to differences in product size, the ratio must be altered when used with the high-throughput primer set).

### Validation of ribosome profiling libraries

We used the Qubit dsDNA High-Sensitivity kit (Life Technologies) to quantify libraries prior to pooling. Libraries were evaluated for the presence of primer and secondary peak with the High-Sensitivity Bioanalyzer DNA chip (Agilent Technologies). In order to fully remove primers and to reduce the contribution of the aforementioned no-insert secondary peak, some libraries require an additional round of 1.2× or 1.0× AMPure XP bead cleanup. Sequencing was performed on a NextSeq 500 desktop sequencer with a 75 cycle high-output kit (Illumina). We obtained between 20 and 50 million demultiplexed, pass-filtered, single-end reads for each sample.

### Bioinformatic analysis of ribosome profiling and RNA-Seq libraries

Each read contains a G-rich region from terminal transferase activity, followed by a ribosome footprint and a poly(A) tail. The first 5 and last 20 bases of each read were removed with fastx_trimmer from the FASTX Toolkit. Because the poly(A) tail can appear at different points in the read, stretches of “AAAAAAAA” at the 3′ end of reads were removed with fastx_clipper; reads shorter than 15 bases after trimming and clipping were discarded. Contaminating rRNA reads were removed by mapping all reads to a rRNA reference library with Bowtie2, allowing for one error and outputting reads which did not align [11]. Reads which did not map to the rRNA reference were aligned to the genome and transcriptome with TopHat2 without looking for novel junctions. Following mapping, read counting was performed with HTSeq set in interstrict mode. We obtained between four and ten million reads uniquely mapped to the CDS per ribosome profiling sample. RNA-Seq data were sequenced and analyzed as previously reported [10]. We obtained between nine and ten million reads uniquely mapped to the CDS per RNA-Seq sample and 17–19 million reads uniquely mapped to exons.

### Calculation of unique fragments

The number of unique fragments was calculated for both methods of ribosome profiling with Picard Tools downloaded from the Broad Institute. Picard Tools was used in MarkDuplicates mode and was run using files downsampled from the original.bam file output from TopHat that was previously generated for each sample. Downsampling was performed with fastq-sample from the fastq-tool suite. Following sorting and indexing with SamTools, the number of unique fragments was determined with Picard Tools.

### Analysis of translational activity and RiboTag enrichment

To analyze differential translation efficiency between the control and AZD-treated samples, we used the recently reported RiboDiff algorithm with the CDS-mapping RNA-Seq and ribosome profiling reads as input [38]. RiboTag enrichment scores were calculated from two RiboTag IP experiments and two homogenate experiments. RiboTag enrichment scores were calculated for each gene by first normalizing counts found in RiboTag and homogenate samples by size factors generated from DESeq2. Following normalization, enrichment scores were calculated by dividing normalized RiboTag counts by normalized homogenate counts.

Translation efficiency was also calculated on a per-sample basis by normalizing ribosome profiling and RNA-Seq counts by size factors from DESeq2 and dividing ribosome profiling counts by RNA-Seq counts. We thresholded downstream analyses by removing genes that had less than 37 counts in ribosome profiling and RNASeq data. When the TE of both samples in a group was used, the threshold was increased to 75 counts.

### Cell type-specific specific lists

We used an RNA-Seq database generated from purified representative cell type populations in order to generate rank lists of cell type-specific genes [20]. We created seven cell type-specific enrichment rank lists, one for each of the seven representative cell types in the database. Enrichment scores for each cell type were calculated for every gene. These scores *E* were calculated for each gene *i* in each cell type *j* and were computed from their cell type-specific RNA expression levels *FPKM*_*ij*_ using the following equation:

$$ E=\frac{FPK{M}_{ij}}{{\displaystyle {\sum}_k}FPK{M}_{ik}}-\frac{1}{2} $$

This resulted in seven cell type-specific enrichment scores between −0.5 and 0.5 for each gene. This value was later recalculated without including newly formed oligodendrocytes as a cell type (in order to improve enrichment among the remaining cell types due to significant overlap between myelinating and newly formed oligodendrocytes). These cell-type enrichment rank lists were later used in gene set enrichment analysis (GSEA) and to define which genes were most associated with specific cell types. Cell type-specific genes were defined as having an enrichment score greater than 0.2.

### Gene set enrichment analysis

In order to determine the role of translational regulation in cell type-specific genes, we performed a GSEA with software downloaded from the Broad Institute [29]. In all instances of GSEA we performed a “classic” GSEA analysis in pre-ranked mode. Gene sets were constructed from previously calculated and thresholded TE values for each sample individually and for combined samples as described above. Between 10,201 and 9904 genes (difference due to previously mentioned thresholding) were ranked based on their TE calculated from untreated RiboTag brains into bins. Equal sized bins spanning 0.75 TE units were constructed around the median and populated with genes based on their TE rank. This was then used as the gene set input for GSEA for each sample.

Cell type-specific enrichment scores, which are described above, were ranked and used to determine if cell type-specific genes were enriched in TE bins. Input to GSEA was a gene set composed of TE values for a given sample (described above) and a rank list composed of the enrichment scores of a single cell type. GSEA was then repeated for the gene set with every cell type rank list. Normalized enrichment scores (NESs) were generated from the GSEA software and then used to generate figures. The statistical significance of differences in TE between cell types was calculated using GSEA. The enrichment scores previously calculated for each cell type were used to generate a new comparison score for each gene *i* in each cell type *k* and *j*:

$$ Es=\frac{E_{ik}}{E_{ij}} $$

Rank lists were then generated for each pairwise combination of cell types composed of calculated comparison scores for each gene. GSEA was run with the same settings as before using the previously generated gene sets based on TE scores. False discovery rate (FDR)-corrected *p* values are plotted in Additional file 3: Figure S3.

### GO analysis

As a secondary means of displaying the cell type-specific translational landscapes we observed, we generated lists of cell type-specific GOs. In order to calculate the enrichment of cell type-specific genes in GOs, a list of 1400 GOs taken from the iPAGE database [39] was used to create gene sets where each set was a single ontology. NES for the enrichment of cell type-specific genes in individual ontologies were produced using this gene set in conjunction with previously generated rank lists comprised of enrichment scores (one for each cell type). A GO was defined as being enriched in an individual cell type if the NES for that cell type was at least three units higher than the next highest NES for that GO. Median TE was calculated for genes within enriched ontologies and plotted.

### 5′ UTR analysis

The number of ribosome profiling and RNA-Seq reads mapped to the 5′ UTR were counted with HTSeq-counts set to region-interstrict mode for each matched sample. Cell type-specific genes were defined for this analysis as having a previously calculated enrichment value greater than 0.2. The fraction of cell type-specific genes with 5′ UTR ribosomal density was calculated as the percentage of cell type-specific genes with at least one ribosomal footprint in the 5′ UTR region. Upstream AUG sequences were identified with a custom python script and defined as any AUG sequence found within the 5′ UTR region of a gene in genes with 5′ UTR density. The median TE was calculated for cell type-specific genes as well as for the subgroups of cell type-specific genes with 5′ UTR density and containing uAUG and genes containing 5′ UTR density without uAUG. The weighted average of 5′ UTR length for each gene was calculated using isoform abundance information from Cufflinks. Cufflinks was quantified against a reference transcript annotation and otherwise run with default settings. GC content of 5′ UTRs was calculated in the same manner using isoform abundance information from Cufflinks. Genes were sorted into bins defined by GC content and length and the median TE was calculated. The significance of the change in TE due to 5′ UTR GC content and 5′ UTR length was calculated using the Mann–Whitney U test.

## Abbreviations

cDNA, complementary DNA; CDS, coding sequence; GO, gene ontology; GSEA, gene set enrichment analysis; HA, hemagglutinin; IP, immunoprecipitation; mTOR, mammalian target of rapamycin; NES, normalized enrichment score; OPC, oligodendrocyte precursor cell; ORF, open reading frame; PBS, phosphate-buffered saline; PCR, polymerase chain reaction; qPCR, quantitative PCR; RT, reverse transcription; TE, translation efficiency; TOP, terminal oligopyrimidine; uORF, upstream open reading frame; UTR, untranslated region

## Additional files

Additional file 1: Figure S1. Sensitivity of conventional and ligation-free strategies. Ligation-free and conventional libraries were generated from a serially diluted 34-base RNA oligonucleotide and analyzed via Bioanalyzer following an equal number of PCR cycles for each library. All ligation-free library preparations except for the 0.01 ng sample were loaded onto the Bioanalyzer at a 1:10 dilution to avoid saturating the detector at high concentrations. Detectable libraries were successfully generated for all concentrations using the ligation-free method but could only be generated using conventional methods for the 100- and 10-ng inputs. (PDF 665 kb) Additional file 2: Figure S2. Highly translated genes identified by ligation-free ribosome profiling are shifted to heavier polysomes. qPCR was performed with five probes on fractions isolated from a polysome profile from left frontal lobe brain tissue. **a** Genes found to be highly translated in ribosome profiling data, Snap-25 and Syt1, were found to be shifted to heavier polysomes; fractions 8 and 9. Genes found to be lowly translated, Tgfb1, Trpv6, and Pkd-1, were found to be most concentrated in lighter polysomes, fractions 4 and 5. **b** The polysome profile denotes from which portion of the profile fractions were obtained. (PDF 509 kb) Additional file 3: Figure S3. Comparison of ligation-free ribosome profiling and RNA-Seq to protein abundances measured by mass spectrometry. RNA-Seq and ligation-free ribosome profiling data from this experiment were plotted against proteomics data from a mouse of the same age and similar background. **a** RNA-Seq data plotted against whole brain mass spectrometry protein abundance are correlated with r = 0.52 and r^2^ = 0.27. **b** Ligation-free ribosome profiling data plotted against whole brain mass spectrometry protein abundance are better correlated than in **a** with r = 0.60 and r^2^ = 0.36. (PDF 1606 kb) Additional file 4: Figure S4. False discovery rate (FDR)-corrected *p* values for pairwise comparisons of each cell type at each TE bin for the heatmaps shown in Fig. 2b computed by GSEA. (PDF 874 kb) Additional file 5: Table S1. List of cell type-specific gene ontologies and their median TEs. (XLSX 13 kb) Additional file 6: Figure S5. We sacrificed mice 1 h after oral administration of AZD-8055 and performed western blot analysis on homogenized brain tissue. Administration of AZD-8055 in a Camk2a-RiboTag mouse decreases mTOR activity as detected by phosphorylation of Rps6. Phosphorylated Rps6 levels were compared with Rps6 and β-actin levels for vehicle, 20 mg/kg AZD-8055, and 100 mg/kg AZD-8055 treatments. *LE* long exposure. (PDF 884 kb) Additional file 7: Table S2. Table of genes altered following AZD-8055 treatment with *p*_*adj*_ values <0.05 and fold change >2. (XLSX 11 kb)
