# Supplementary figures and images for: Ligation-free ribosome profiling of cell type-specific translation in the brain
Source: Genome Biol. 2016 Jul 5;17:149. doi: 10.1186/s13059-016-1005-1 (PMC4934013; doi:10.1186/s13059-016-1005-1)

Conventional  
Library Preparation

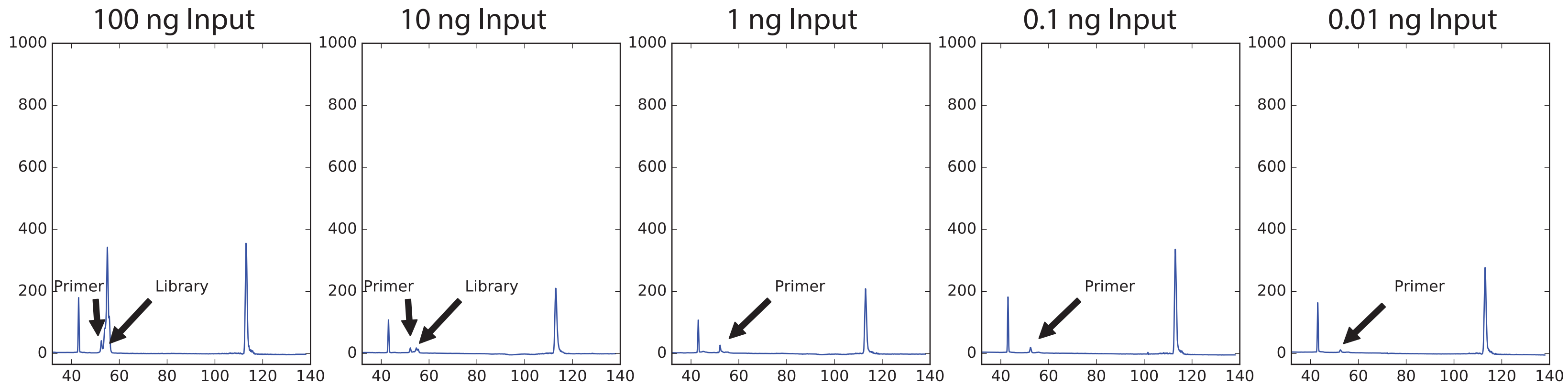

Ligation-Free  
Library Preparation

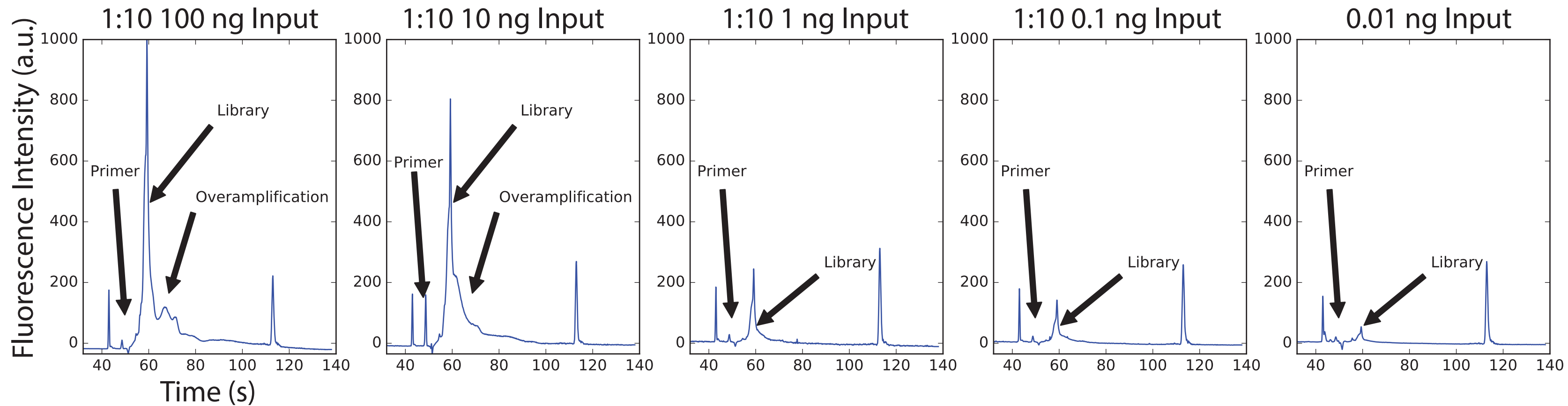

Supplement: Additional file 1: Figure S1. — Sensitivity of conventional and ligation-free strategies. Ligation-free and conventional libraries were generated from a serially diluted 34-base RNA oligonucleotide and analyzed via Bioanalyzer following an equal number of PCR cycles for each library. All ligation-free library preparations except for the 0.01 ng sample were loaded onto the Bioanalyzer at a 1:10 dilution to avoid saturating the detector at high concentrations. Detectable libraries were successfully generated for all concentrations using the ligation-free method but could only be generated using conventional methods for the 100- and 10-ng inputs. (PDF 665 kb) [file 13059_2016_1005_MOESM1_ESM.pdf]

A

## qPCR Gene Abundance Per Fraction

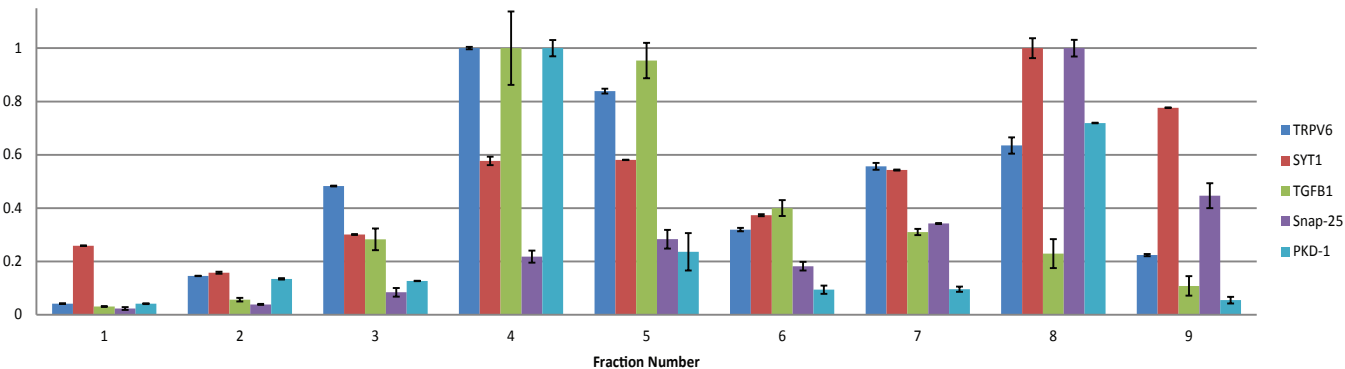

B

## Polysome Profile

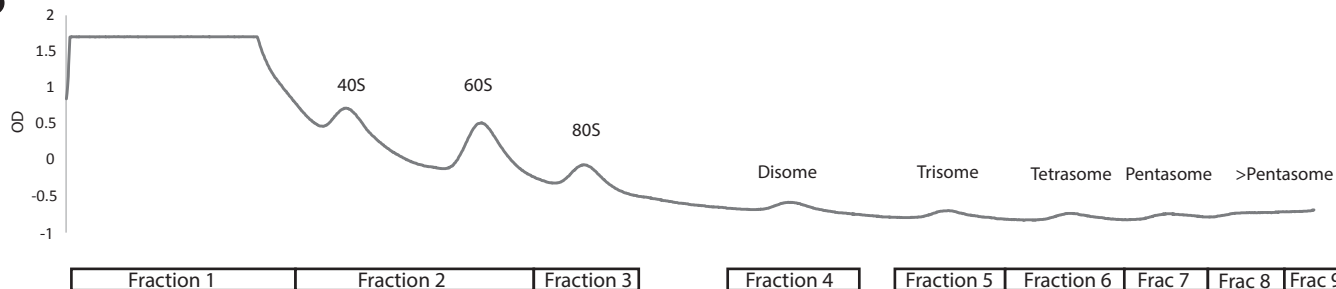

Supplement: Additional file 2: Figure S2. — Highly translated genes identified by ligation-free ribosome profiling are shifted to heavier polysomes. qPCR was performed with five probes on fractions isolated from a polysome profile from left frontal lobe brain tissue. a Genes found to be highly translated in ribosome profiling data, Snap-25 and Syt1, were found to be shifted to heavier polysomes; fractions 8 and 9. Genes found to be lowly translated, Tgfb1, Trpv6, and Pkd-1, were found to be most concentrated in lighter polysomes, fractions 4 and 5. b The polysome profile denotes from which portion of the profile fractions were obtained. (PDF 509 kb) [file 13059_2016_1005_MOESM2_ESM.pdf]

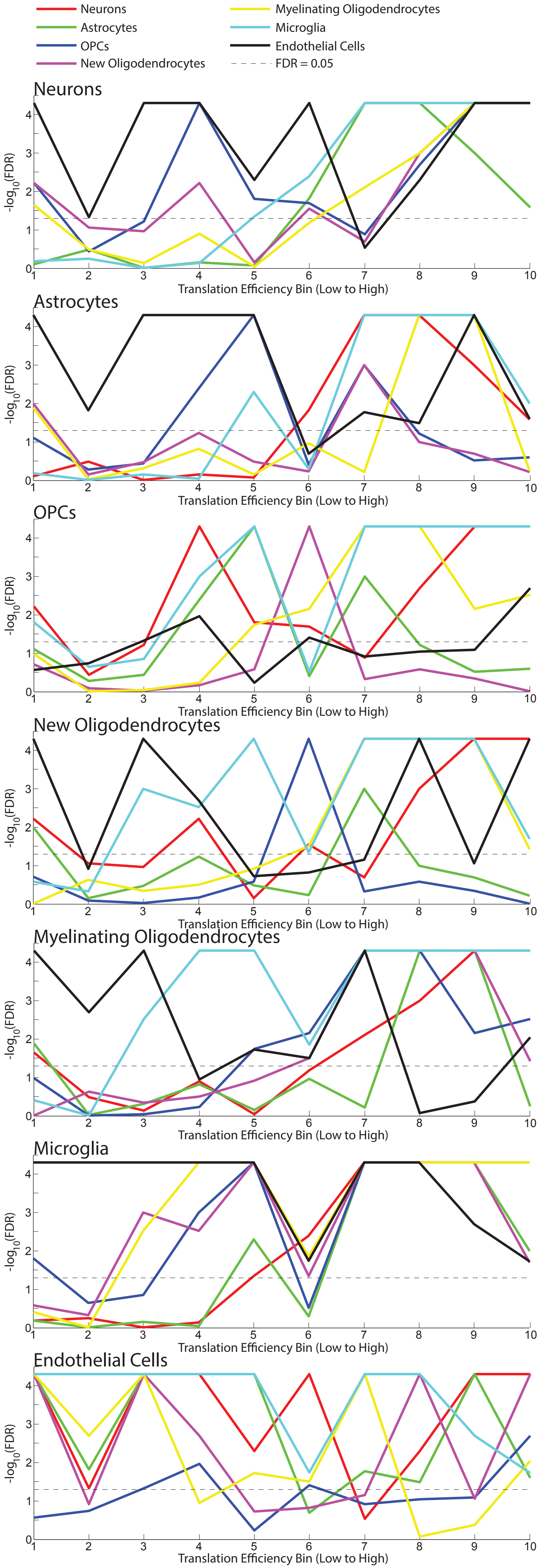

Supplement: Additional file 4: Figure S4. — False discovery rate (FDR)-corrected p values for pairwise comparisons of each cell type at each TE bin for the heatmaps shown in Fig. 2b computed by GSEA. (PDF 874 kb) [file 13059_2016_1005_MOESM4_ESM.pdf]

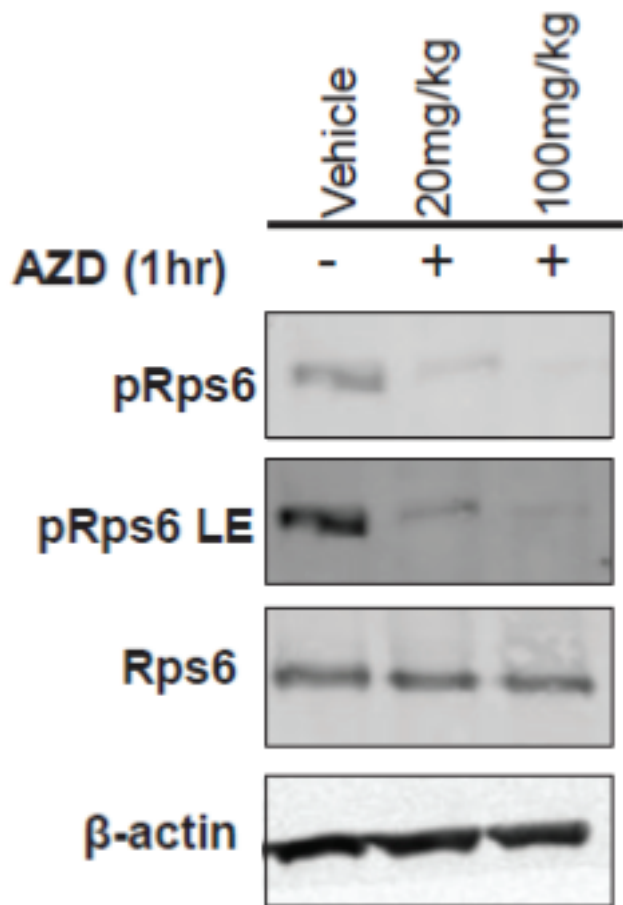

Supplement: Additional file 6: Figure S5. — We sacrificed mice 1 h after oral administration of AZD-8055 and performed western blot analysis on homogenized brain tissue. Administration of AZD-8055 in a Camk2a-RiboTag mouse decreases mTOR activity as detected by phosphorylation of Rps6. Phosphorylated Rps6 levels were compared with Rps6 and β-actin levels for vehicle, 20 mg/kg AZD-8055, and 100 mg/kg AZD-8055 treatments. LE long exposure. (PDF 884 kb) [file 13059_2016_1005_MOESM6_ESM.pdf]
