# Supplementary material for: Ligation-free ribosome profiling of cell type-specific translation in the brain
Source: Genome Biol. 2016 Jul 5;17:149. doi: 10.1186/s13059-016-1005-1 (PMC4934013; doi:10.1186/s13059-016-1005-1)
Supplement: Additional file 3: Figure S3. — Comparison of ligation-free ribosome profiling and RNA-Seq to protein abundances measured by mass spectrometry. RNA-Seq and ligation-free ribosome profiling data from this experiment were plotted against proteomics data from a mouse of the same age and similar background. a RNA-Seq data plotted against whole brain mass spectrometry protein abundance are correlated with r = 0.52 and r2 = 0.27. b Ligation-free ribosome profiling data plotted against whole brain mass spectrometry protein abundance are better correlated than in a with r = 0.60 and r2 = 0.36. (PDF 1606 kb) [file 13059_2016_1005_MOESM3_ESM.pdf]

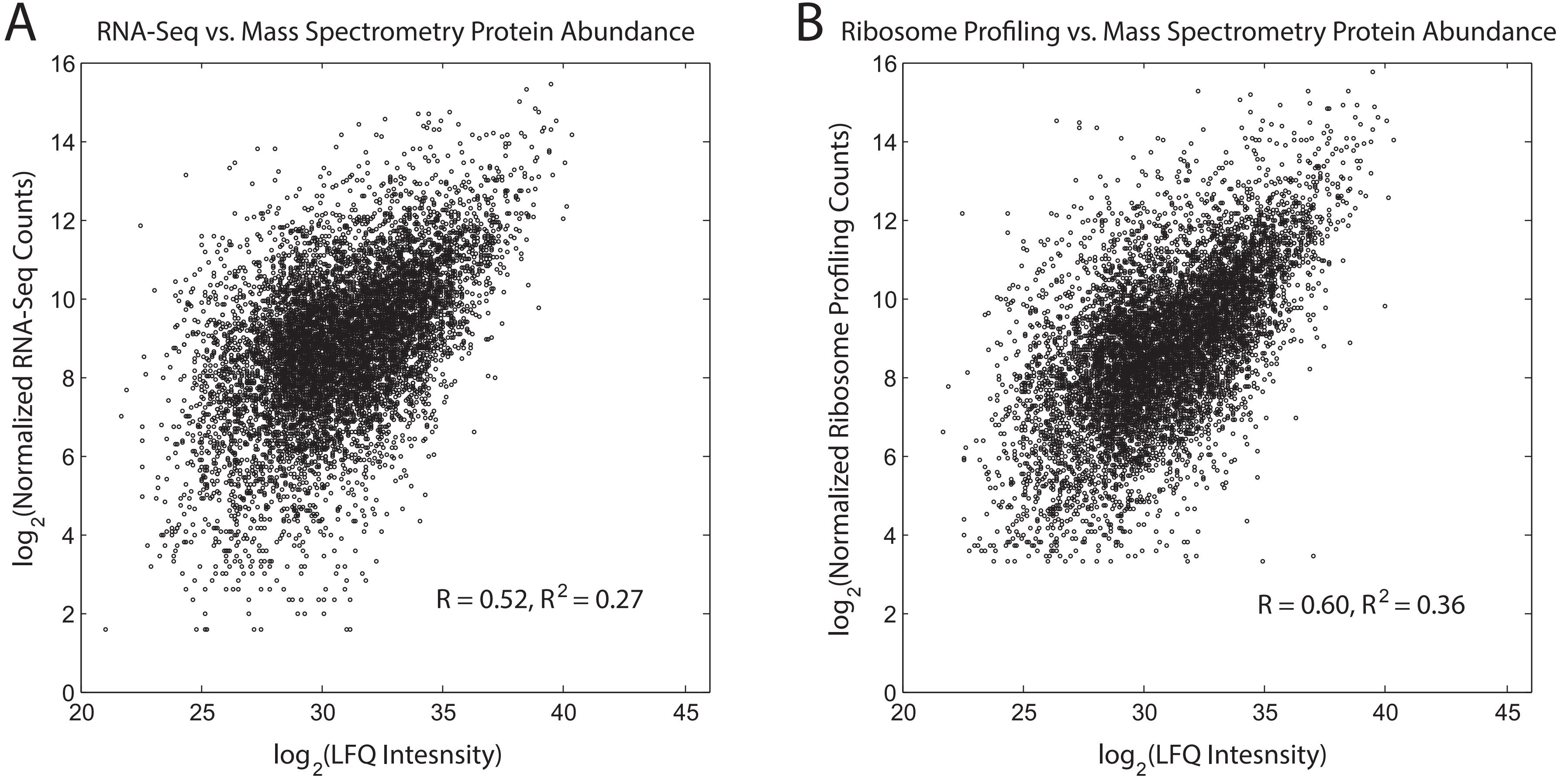
A
RNA-Seq vs. Mass Spectrometry Protein Abundance
log2(Normalized RNA-Seq Counts)
log2(LFQ Intesnsity)
R = 0.52, R2 = 0.27
B
Ribosome Profiling vs. Mass Spectrometry Protein Abundance
log2(Normalized Ribosome Profiling Counts)
log2(LFQ Intesnsity)
R = 0.60, R2 = 0.36
